# Dengue Epidemiology in Qatar from 2013–2021: A Retrospective Study

**DOI:** 10.3390/tropicalmed7110329

**Published:** 2022-10-25

**Authors:** Elmoubashar Abd Farag, Shariq Jaffrey, Faisal Daraan, Maha Hammam M. A. Al-Shamali, Fahmi Y. Khan, Peter V. Coyle, Francis Schaffner, Hamad Eid Al-Romaihi, Mohammed Al-Thani, Devendra Bansal

**Affiliations:** 1 Health Protection and Communicable Diseases, Public Health Department, Ministry of Public Health, Doha P.O. Box 42, Qatar; 2 Department of Medicine, Hamad General Hospital, Hamad Medical Corporation, Doha P.O. Box 3050, Qatar; 3 Department of Virology, Hamad Medical Corporation, Doha P.O. Box 3050, Qatar; 4 Francis Schaffner Consultancy, Lörracherstrasse 50, 4125 Riehen, Switzerland

**Keywords:** dengue, epidemiology, State of Qatar

## Abstract

(1) Background: Qatar does not have any indigenous cases of dengue; however, the influx of immigrants from dengue endemic countries, the environment, and climate suitability for Aedes vector mosquitoes suggest a potential risk for local transmission. In this study, we investigated various demographic factors to determine the epidemiological features of dengue in Qatar. (2) Methods: In the present retrospective study, we reviewed dengue notification data received at the national surveillance system, Ministry of Public Health, Qatar, between January 2013, and December 2021, and we analyzed the incidence of the dengue disease burden to identify factors that could contribute to the dissemination of the disease in Qatar. (3) Results: A total of 166 dengue fever seropositive cases were recorded during the study period in Qatar. The mean incidence was estimated to be 0.7/100,000 population, which increased from 0.7/100,000 in 2013 to 1.5/100,000 in 2019. The majority of the cases were male, between 20–50 years of age and notified during the hot months (June-September). Most of the patients had fever without hemorrhagic manifestations. There were no dengue related deaths during 2013–2021. (4) Conclusion: Dengue fever occurred more frequently among men than women, and its incidence is low among Qatari nationals. The presence of the most efficient vector, *Aedes aegypti*, in Qatar, if confirmed, poses a risk of local outbreaks. Therefore, regular vector surveillance is needed to assess the distribution, biting habits and abundance of vector mosquito species and the risk for mosquito-borne diseases.

## 1. Introduction

Dengue fever is a mosquito-borne arbovirus disease with a major impact on public health in the tropics and sub-tropics [1]. It is one of the twenty diseases on the WHO list of neglected tropical disease (NTD) roadmap [2]. Over the last two decades, the incidence rate for dengue fever has increased 30-fold around the world. According to the World Health Organization (WHO), an estimated 3.9 billion people are at risk of dengue virus (DENV) infection in 128 countries. Approximately 100–400 million infections occur each year among which more than 80% of cases are generally mild and asymptomatic [1].

All four serotypes of DENV form a spectrum of clinical illnesses ranging from classical dengue fever to severe and potentially fatal complications known as dengue hemorrhagic fever (DHF) and dengue shock syndrome (DSS) [3,4,5]. DHF continues to be a major public health problem with a steady rise in outbreaks around different parts of the world in recent decades, with cases increasing from 505,430 to 2.4 million between 2000 and 2010 [6]. However, in Qatar, dengue fever is uncommon, with cases being imported from endemic areas through infected travelers. Recently, lower antibodies of DENV were detected in residents of Qatar, as well as in Middle East and North African (MENA) nationals compared to Asian nationals, suggesting a lower burden of DENV disease in the MENA region [7].

Early diagnosis of DENV infection is important for proper treatment of DHF and DSS to avoid a fatal outcome. In December 2015, a recombinant, live attenuated, tetravalent chimeric vaccine, Dengvaxia (CYD-TDV), was initially licensed in Mexico, and has now been licensed for use in over 10 dengue-endemic countries for individuals 9 to 45 years of age [8]. Nevertheless, an increased risk of severe dengue requiring hospitalization was associated with vaccination of DENV seronegative individuals [9]. Currently, several dengue vaccine candidates are in an advanced stage of development and have entered phase 3 clinical testing [10,11].

The *Aedes aegypti* mosquito species is the primary vector for dengue, followed by *Ae. albopictus*, a secondary dengue vector, which can also develop in temperate regions, unlike *Ae. aegypti* [12,13,14]. Peak DENV infection occurs after periods of intense rainfall due to increased multiplication of the vector mosquito populations [13]. The high temperatures in Qatar may limit the proliferation of mosquitoes; however, extensive construction and prolonged use of air conditioning has altered the breeding habits of the vector, leading to their increased prevalence in Gulf Cooperation Council (GCC) countries [15,16,17]. 

The State of Qatar is rapidly developing, with a high number of expats migrating from dengue endemic countries. Additionally, Qatar has one of the busiest international airports in the world, serving thousands of passengers who transit through the country every day. These factors can easily facilitate the spread of dengue, yellow fever, Zika and other mosquito borne flavivirus via local vectors [7]. Moreover, Qatar is set to host the FIFA World Cup 2022, which is expected to draw in a surge of visitors, many of whom could be from dengue endemic countries. The present study aimed to estimate the incidence of dengue fever in Qatar to impede potential spread of the disease in preparation for the FIFA World Cup 2022. 

## 2. Materials and Methods

This study was a retrospective review of all dengue fever cases reported at the national surveillance and outbreak unit at the Ministry of Public Health (MoPH), Qatar, between January 2013 and December 2021. Hamad Medical Corporation (HMC) is the main secondary and tertiary care provider in the country; it caters to the population of Qatar and epidemiologically represents both locals and expatriates in the country. A diagnosis of dengue fever was established by measurement of dengue specific IgM and IgG using commercially available kits (Euroimmun, Lübeck, Germany) and confirmed by syndromically grouped real-time PCR kits (Fast Track Diagnostics, Esch-sur-Alzette, Luxembourg). In the present study, dengue cases were reported from all the healthcare facilities including governmental, semi-governmental and private health institutions across the country. This study also collected demographical information such as age, gender, nationality and travel history. 

### 2.1. Dengue Case Management

In Qatar, the management of vector borne disease cases is centralized under HMC and typically seen in the emergency department and triaged according to case definition and complications. Management of cases at HMC is supported by consistently maintaining adequate intravascular volume with the use of intravenous fluids and blood transfusion in cases of bleeding complications. All patients diagnosed with dengue were treated as per the current guidelines at HMC. The travel clinic at HMC provides standardized advise to travelers visiting dengue endemic areas on how to avoid and prevent mosquito bites [18]. 

### 2.2. Data Analysis

Data were collected and entered in Microsoft Excel. A cross-section analysis for different variables was performed using StataCorp, 2011 (Stata Statistical Software: Release 12. College Station, TX, USA: StataCorp LP). Data were reported as means ± standard deviation (SD) for quantitative variables, while qualitative variables were described as numbers, percentages and case incidence per 100,000 population. Multivariate analysis using logistic regression was performed and *p* value < 0.05 considered to be statistically significant. 

## 3. Results

**Figure 1:**
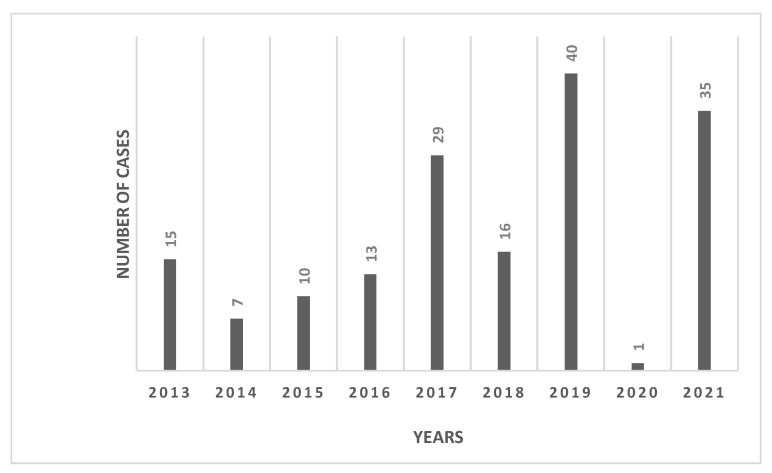
Distributions of dengue fever cases in Qatar, 2013–2021.

A total of 166 seropositive cases were included in this study over the nine-year period from 2013–2021. The mean incidence was estimated to be 0.7/100,000 population. Figure 1 shows a trend of a gradual rise in numbers over the years which culminates with the highest number of 40 cases recorded in year 2019 (incidence rates increased from 0.7/100,000 in 2013 to 1.5/100,000 in 2019). The other two peaks were 35 and 29 cases in 2021 and 2017, respectively. Only one case was reported in 2020; this may have been due to travel restriction and under-reporting as the COVID-19 pandemic took precedence in terms of public health resources worldwide.

**Figure 2:**
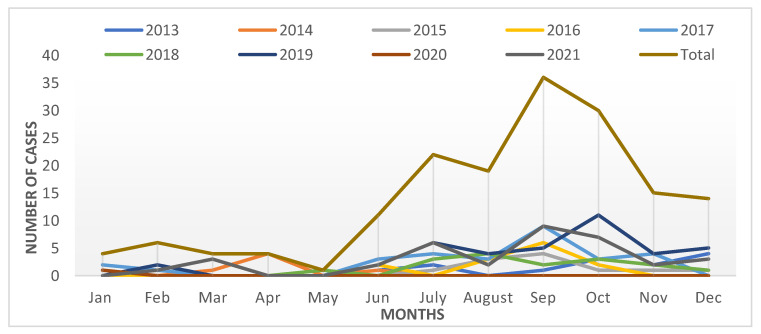
Monthly trends of reported dengue cases between 2013 and 2021 in Qatar.

The epidemiological features of the DENV cases (Table 1) show that most cases were males (76%), reflecting the high number of single male workers, including both blue- and white-collar workers, who form much of the immigrant population in Qatar. The mean age of the cases was 32.9 ± 12, and most of them were aged between 20 to 50 years (79%). The incidence rate among this age group was 7/100,000 population. Among nationalities, the majority of the cases were non-Qatari (98%), particularly from southeast Asian countries. In addition, almost 64.4% of cases were reported between July and October (Figure 2). All patients were symptomatic, and fever was found to be ubiquitous, with 99% of cases exhibiting this symptom. None of the cases had hemorrhaging, which indicates the absence of severe dengue in Qatar. With regard to the frequency distribution of the seropositivity of cases, the majority were IgM antibodies (130/166, 78%), followed by IgG (96/166, 58%) and both IgM and IgG positive (60/166, 36%). None of the studied epidemiological factors had any significant association with seropositivity.

## 4. Discussion

Qatar is home to a large number of immigrants, making up approximately 88% of the total population of 2.8 million, among which Indian nationals form the largest population cluster, followed by other Southeast Asian nations, all of which are dengue endemic [19]. The gradual rise in the number of cases during 2013–2021 reflects the influx of labor workers as Qatar embarks upon mega construction projects. However, it was noteworthy that only one case was reported in 2020; this singular oddity could be explained through travel restrictions and shifting priorities during the COVID-19 pandemic, when healthcare facilities and surveillance resources were rerouted to give priority to addressing that situation. Additionally, in this study, the immigrant worker contribution to case load distribution is further reflected in the age group and gender distribution, as most of migrants in the country are single, young, male, blue collar workers. The incidence of disease among Qatari nationals is very low (2%) and is likely due to travel exposure, as all of them had travel history to endemic countries. Furthermore, dengue is known to be a frequent cause of morbidity among international travelers returning from south and southeast Asia and Latin America [20]. However, in the present study, all patients had travel history to dengue endemic countries, and no dengue related deaths were reported during 2013–2021.

The seroprevalence study of dengue among Qatar residents revealed the highest rate of infection among Philippines nationals (95.8%), followed by expats from India (62.5%), Sudan (48.5%), Yemen (24.2%) and Pakistan (20.0%) [7]. In contrast, the exposure of dengue among Qatar and other MENA nationals was lower compared to Asian nationals, suggesting a lower burden of the disease in this region. However, the presence of antibodies in most MENA nationals suggests that there is a need to better understand the epidemiology of this virus and disease burden among this population [7]. In the present study, the majority of the dengue fever cases were among south and southeast nationals who had IgM antibodies. Interestingly, all dengue cases among African nationals were found to be IgM positive. However, logistic regressions for IgM and IgG showed no significant association with any of the independent variables.

The disease has a wide presentation spectrum, ranging from mild viral illness to life threatening bleeding and death. At present, Qatar does not have any reported cases of severe dengue with hemorrhagic fever. Nevertheless, in non-endemic countries, local outbreaks are triggered by the presence of potential dengue vectors along with other potential factors such as an increase of international travel and imported cases [21,22,23]. In Qatar, *Ae. aegypti* was reported in 1999 by the Armed Forces Pest Management Board; however, in our recent surveys, it was not found, suggesting no risk of local transmission in the country [15,24,25,26].

Another factor that could potentially exacerbate the spread of dengue is the weather, which has been considerably influenced by global climate change. These changes might lead to increased vector survival, reproduction and biting rate, which would, in turn, lead to longer transmission seasons [27,28]. Moreover, climate change affects the environment and human health and has social and economic implications. Qatar experiences a much harsher climate, with generally high temperatures during summer months and variable humidity, which makes for viable environmental conditions for disease transmission [19]. From June to August, the average temperatures in the country reached between 35 to 37 °C. However, the maximum temperature during summer can reach well beyond 45 °C. The humidity index in Qatar typically tends to be high, averaging in the range of 45% to 65% during summer (August and September), fall, and most of the winter, lasting until the following February. Afterward, the average monthly humidity decreases to 26% (June), and then increases again during the summer [19]. Recently, we found that higher temperatures and lesser humidity enhance the survival and reproduction rates of *Anopheles* and *Aedes* mosquitoes. However, the likelihood of detecting *Aedes* mosquitos increases as humidity decreases, but only to a certain point, before dropping again. Temperatures between 35 and 40 °C and relative humidity levels between 35% and 45% are ideal for *Aedes* mosquitoes (Unpublished data).

Regarding the seasonal distribution of dengue cases, it generally peaks in July to September in tropical and sub-tropical countries [1]. The prevalence of dengue fever in southeast Asian countries is high during May to November [1]. In our study, it was observed that cases gradually increased from July to October (64.4%). This rise resulted from migrant workers and/or expatriates returning to Qatar during these months after their summer vacations in their respective home countries. This trend remains consistent with previous reports from GCC and European countries [29,30,31,32]. 

It is a known fact that species of *Ae. mosquitoes* have adapted to urbanization and habitat changes. *Ae. aegypti* typically does very well in heavily urbanized environments, largely due to its reproductive strategy of exploiting small volumes of water in manmade containers around housing as larval habitats [33]. In places with similar climatic conditions to Qatar, air-conditioning ducts and industrial areas with stagnant water have become the preferred breeding grounds for the *Aedes* mosquitoes [15,27,34]. As the majority of the population in Qatar is expats, it is vital for the country to have active surveillance for the most efficient vectors, *Ae. aegypti* and *Ae. albopictus*, given that vector control remains the primary strategy for outbreak prevention and control, especially considering the recent outbreaks in neighboring countries [17,35,36,37,38,39]. Vaccination programs have shown limited success and are not cost effective to counter dengue. Effective vector control is the cornerstone of strategy development to prevent or control potential outbreaks. As per the WHO’s NTD control 2021–2030 roadmap, vector control and effective surveillance are the most crucial ways to successfully manage vector-borne diseases [2]. As such, to strengthen vector management and prevent VBD outbreaks during the 2022 FIFA World Cup, several recommendations have been published [40]. Apart from a large immigrant population, Qatar is also a major travel hub and is soon to host FIFA World Cup 2022, which is expected to bring thousands of visitors from all over the world. The event will be held during the winter season, when the temperature is much cooler. To prevent any large scale spread of dengue, early planning on vector surveillance and control is essential [30]. This is also applicable to other *Aedes* vector borne disease like chikungunya and Zika and *Anopheles* vector-borne disease such as malaria. Previous experiences of world cup hosting nations such as South Africa and Brazil can be used for effective strategy planning and implementation. Increased awareness of healthcare staff with low thresholds of suspicion for VBDs should be instituted for timely detection and response, thereby preventing an outbreak. Vector control, with mosquito free zones around stadiums, as implemented in South Africa during the 2010 FIFA World Cup, can be used as a model to protect travelers and residents during the event. 

### Study Limitations

Our study has some limitations that should be considered when interpreting the results. First, this was a retrospective study; therefore, we were not able to follow up patients as we relied primarily on patient notification reports in the surveillance and vaccine electronic system at MoPH. Second, there was a lack of data related to blood test results and disease outcomes. Third, since no asymptomatic infections were recorded in the MoPH’s electronic surveillance and vaccine system, incidence rate estimates should be interpreted with caution.

## 5. Conclusions

Our study demonstrated that dengue remains limited in Qatar, with the low numbers being attributed to expat influx and all cases being related to travel to disease endemic countries. However, the seropositivity and presence of potential vectors indicates possible risk of dengue transmission. Qatar has a robust public health infrastructure and surveillance mechanism in place, allowing it to be vigilant for potential health threats. In the long term, however, it is imperative to develop effective and comprehensive surveillance and management systems for mosquito transmitted diseases, considering that the FIFA World Cup is expected to draw a large number of visitors. Further epidemiological and entomological surveillance and climate change studies are required to improve early detection and response. 

## Figures and Tables

**Table 1:** Demographic and clinical characteristics of 166 DENV serology confirmed cases in Qatar, 2013–2021.

Variables	TOTAL *n* = 166 (%)	Dengue IgM Positive *n* = 130 (%)	Dengue IgG Positive *n* = 96 (%)	Dengue IgM & IgG Positive *n* = 60 (%)
Sex				
Female	40 (24)	32 (25)	25 (26)	17 (28)
Male	126 (76)	98 (75)	71 (74)	43 (72)
Age				
<10	6 (4)	6 (5)	1 (1)	1 (2)
10–20	16 (10)	14 (11)	7 (7)	5 (8)
21–30	44 (27)	33 (25)	24 (25)	13 (22)
31–40	58 (35)	44 (34)	37 (39)	23 (38)
41–50	29 (17)	23 (18)	17(18)	11 (18)
>50	13 (7)	10 (8)	10(10)	7 (12)
Nationality				
South & Southeast Asian ^#^	146 (8)	112 (86)	87 (91)	53 (88)
African Region ^$^	7 (4)	7 (5)	4 (4)	4 (7)
Others ^@^	13 (8)	11 (8)	5 (5)	3 (5)
Symptoms				
Fever	165 (99)	129 (99)	96 (100)	60 (100)
Headache	46 (28)	332 (25)	30 (31)	16 (27)
Joint Pain	39 (23)	29 (22)	27 (28)	17 (28)
Muscle Pain	45 (27)	34 (26)	30 (31)	19 (32)
Rash	16 (10)	13 (10)	11 (11)	8 (13)
Fatigue	16 (10)	12 (9)	12 (13)	8 (13)
Vomiting	11 (6.61)	6 (6)	7 (7)	4 (7)
Others	14 (8)	7 (5)	12 (12)	5 (8)

^#^ South and southeast countries: Indian (89), Filipino (12), Sri Lankan (12), Pakistani (12), Bangladeshi (10), Nepalese (5), Indonesian (4), and Chinese (1); ^$^ African Region: Nigerian (2), and one of each Kenyan, South African, Sudanese, and Ugandan; ^@^ Cuban (3), Qatari (3) Australian (2), and one each of American, British, Egyptian, Italian, Portuguese, Syrian, Tunisian.

## Data Availability

Not applicable.
